# Bidirectional associations of accelerometer-derived physical activity and stationary behavior with self-reported mental and physical health during midlife

**DOI:** 10.1186/s12966-021-01145-4

**Published:** 2021-06-06

**Authors:** Bethany Barone Gibbs, Barbara Sternfeld, Kara M. Whitaker, Jennifer S. Brach, Andrea L. Hergenroeder, David R. Jacobs, Jared P. Reis, Stephen Sidney, Daniel White, Kelley Pettee Gabriel

**Affiliations:** 1 Department of Health and Human Development, University of Pittsburgh, 32 Oak Hill Court, Room 220, Pittsburgh, PA 15216 USA; 2 Kaiser Permanente Northern California, Oakland, USA; 3 University of Iowa, Iowa, USA; 4 University of Minnesota, Minneapolis, USA; 5 National Heart Lung and Blood Institute, Bethesda, USA; 6 University of Delaware, Newark, USA; 7 University of Alabama at Birmingham, Birmingham, USA

**Keywords:** Physical activity, Sedentary behavior, Self-rated health, Cohort study

## Abstract

**Background:**

Moderate-to-vigorous intensity physical activity (MVPA) is associated with favorable self-rated mental and physical health. Conversely, poor self-rated health in these domains could precede unfavorable shifts in activity. We evaluated bidirectional associations of accelerometer-estimated time spent in stationary behavior (SB), light intensity physical activity (LPA), and MVPA with self-rated health over 10 years in in the CARDIA longitudinal cohort study.

**Methods:**

Participants (*n* = 894, age: 45.1 ± 3.5; 63% female; 38% black) with valid accelerometry wear and self-rated health at baseline (2005–6) and 10-year follow-up (2015–6) were included. Accelerometry data were harmonized between exams and measured mean total activity and duration (min/day) in SB, LPA, and MVPA; duration (min/day) in long-bout and short-bout SB (≥30 min vs. < 30 min) and MVPA (≥10 min vs. < 10 min) were also quantified. The Short-Form 12 Questionnaire measured both a mental component score (MCS) and physical component score (PCS) of self-rated health (points). Multivariable linear regression associated baseline accelerometry variables with 10-year changes in MCS and PCS. Similar models associated baseline MCS and PCS with 10-year changes in accelerometry measures.

**Results:**

Over 10-years, average (SD) MCS increased 1.05 (9.07) points, PCS decreased by 1.54 (7.30) points, and activity shifted toward greater SB and less mean total activity, LPA, and MVPA (all *p* < 0.001). Only baseline short-bout MVPA was associated with greater 10-year increases in MCS (+ 0.92 points, *p* = 0.021), while baseline mean total activity, MVPA, and long-bout MVPA were associated with greater 10-year changes in PCS (+ 0.53 to + 1.47 points, all *p* < 0.005). In the reverse direction, higher baseline MCS and PCS were associated with favorable 10-year changes in mean total activity (+ 9.75 cpm, *p* = 0.040, and + 15.66 cpm, *p* < 0.001, respectively) and other accelerometry measures; for example, higher baseline MCS was associated with − 13.57 min/day of long-bout SB (*p* < 0.001) and higher baseline PCS was associated with + 2.83 min/day of MVPA (*p* < 0.001) in fully adjusted models.

**Conclusions:**

The presence of bidirectional associations between SB and activity with self-rated health suggests that individuals with low overall activity levels and poor self-rated health are at high risk for further declines and supports intervention programming that aims to dually increase activity levels and improve self-rated health.

**Supplementary Information:**

The online version contains supplementary material available at 10.1186/s12966-021-01145-4.

## Background

Performing habitual moderate-to-vigorous intensity physical activity (MVPA) and limiting prolonged sedentary behavior are associated with increased life expectancy [1–3]. However, efforts to increase life expectancy should occur in parallel with those focused on optimizing health-related quality of life [4]. Self-rated health provides an easily-measured summary of health-related quality of life as experienced by the individual across domains, e.g. mental and physical [5]. Though high MVPA and low sedentary behavior have been consistently linked to certain mental health outcomes (e.g., reduced depression and anxiety) [6–9] and a decreased incidence of chronic diseases (e.g., diabetes, coronary heart disease, cancer, and physical dysfunction) [1, 3, 9, 10], limited longitudinal studies measure the relationships between MVPA and sedentary behavior with summary measures of self-rated health [11–13]. Few studies use device-based measurement of MVPA and sedentary behavior and even fewer consider the full spectrum of intensity categories (i.e., sedentary behavior, light-intensity physical activity (LPA), and MVPA) or bout durations (i.e., time accumulated in shorter vs. longer bouts of sedentary behavior and MVPA) [2, 14]. Also, most studies are in older adults and there is a dearth of studies during mid-life, which is a critical period for chronic disease and disability development [15].

Little is known about how self-rated mental and physical health might associate with future overall activity levels. We recently demonstrated bidirectional effects between accelerometer-measured activity and stationary behavior (SB) (an estimate of sedentary behavior that can be measured with a waist-worn accelerometer [16]) with obesity, including that greater baseline obesity was associated with increased SB and decreased LPA and MVPA over 10-year follow-up across midlife [17]. Such bidirectionality of effects between SB and activity is also plausible with self-rated health since lower mental and physical health are established barriers to physical activity [18, 19]. This bidirectional possibility is important to study as it may change interpretations of cross-sectional associations and inform clinical and public health strategies for the joint preservation of healthy activity levels and self-rated health.

To address these research gaps, we first examined associations of baseline and 10-year changes in accelerometer-derived estimates across the spectrum of physical activity intensities (SB, LPA, and MVPA) with 10-year changes in self-rated physical and mental health in the Coronary Artery Risk Development in Young Adults (CARDIA) Study [20]. We hypothesized that lower baseline activity levels would be associated with 10-year reductions in self-rated health. Next, in the same cohort, we evaluated the reverse direction by examining associations of baseline and 10-year changes in physical and mental self-rated health with 10-year changes in activity levels. We hypothesized that lower baseline self-rated health would be associated with less favorable 10-year changes in activity levels.

## Methods

### Participants and setting

This analysis was conducted in CARDIA, a multicenter cohort study of the development and determinants of cardiovascular disease beginning in early adulthood (clinicaltrials.gov record NCT00005130) [20]. In 1985–6, CARDIA recruited a biracial (black and white) cohort of 5115 young adults, aged 18–30 years, from field centers in four U.S. cities (Birmingham, Alabama; Chicago, Illinois; Minneapolis, Minnesota; and Oakland, California). CARDIA has followed these participants with in-person exams at least every 5 years thereafter. The current analysis uses data from the year 20 (2005–06) and year 30 (2015–16) follow-up exams, which captured 72 and 71% of the surviving cohort, respectively. Data from the year 20 exam were considered ‘baseline’ and data from the year 30 exam were considered ‘10-year follow-up’. All participants provided informed consent and research procedures were approved by local Institutional Review Boards at each site.

Among those participating in both exams (*n* = 2947), we excluded 2030 participants because they did not participate or were excluded from the CARDIA Fitness Study (2005–6) or the CARDIA Activity Study (2015–6) or had non-compliant wear at either exam. Importantly, the CARDIA Activity Ancillary Study began midway through the exam period, which resulted in missing a portion of potential participants due to timing [21]. Further exclusion of 24 participants with incomplete self-rated health (SF-12) data resulted in a final analytic sample of *n* = 894. Among participants attending both exams, those included vs. excluded in the current analysis were more likely to be white (61.7% vs. 51.3%, *p* < 0.001), female (63.5% vs. 54.8%, *p* < 0.001), and had lower BMI (28.5 kg/m^2^ vs. 29.7 kg/m^2^, *p* < 0.001), but did not differ by age (45.1 years vs. 45.3 years, *p* = 0.224). Included vs. excluded participants had similar scores for mental self-rated health (*p* > 0.05), but had higher self-rated physical health scores at baseline (52.3 vs. 50.9 pts., *p* < 0.001) and 10-year follow-up (50.7 vs. 49.1 pts., *p* < 0.001).

### Measurements

Physical activity was assessed by uniaxial accelerometry (ActiGraph 7164, Pensacola, FL) at the baseline and triaxial accelerometry (ActiGraph wGT3X-BT, Pensacola, FL) at the 10-year follow-up. Participants were instructed to wear the accelerometers for 7 days and during all waking hours (except water activities). Accelerometer counts were exported using ActiLife software and were harmonized across exams using a validation study where both monitors were worn simultaneously by a subset of 87 CARDIA participants during the follow-up exam [22]. This study allowed for the calculation of a calibration factor which divided follow-up wGT3X-BT data (vertical axis data collected at 40 hertz and reintegrated to count data expressed in 60-s epochs) by 1.088 to make it comparable to the baseline data from the ActiGraph 7164 (count data in 60-s epochs). Wear time was calculated as 24 h minus nonwear time. Nonwear time was defined as intervals with 0 counts per minute (cpm) for ≥60 consecutive minutes, but allowing ≤2 min at < 100 cpm [23]. Accelerometry data were considered a valid representation of the seven-day data collection period with ≥4 days of monitoring with ≥10 h/day [24]. One participant was removed due to implausible mean total activity baseline (average > 20,000 cpm) [25].

Daily averages of accelerometry variables, including mean total activity (measured in cpm) and intensity category durations (measured in min/day), were calculated by averaging across valid wear days. Freedson cutpoints were used to classify time spent stationary, i.e., SB (< 100 cpm), LPA (100 to < 1952 cpm), and MVPA (≥1952 cpm) [26]. SB was segmented into short-bout SB (accumulated in < 30-min bouts) and long-bout SB (accumulated in ≥30-min bouts). The 30-min threshold was selected based on research suggesting SB accumulated in bouts ≥30 min is more strongly associated with health outcomes [2, 27] and an expert review concluding that interrupting SB every 30 min is a potentially feasible and health-enhancing behavioral target [28]. Also, reflecting a recent expert call to examine whether MVPA accumulated in bouts of < 10 min is associated with health outcomes in large-sample longitudinal studies [14], MVPA was also segmented into short-bout MVPA (bouts of < 10 min) and long-bout MVPA (bouts of ≥10 min). Long-bout MVPA (e.g., ≥ 10 min) measured by the waist accelerometer was operationalized using a standard approach (bouts of ≥10 min, with allowance for 2 min < 1952 cpm) that acknowledges small interruptions to physical activity that might occur, e.g., briefly pausing at a stop light during a brisk walk or run [14, 26]. Separation of accelerometer-measured activity into intensity x duration categories (long-bout SB, short-bout SB, LPA, short-bout MVPA, and long-bout MVPA) responds to recent calls for longitudinal studies to evaluate the health effects of prolonged SB vs. SB with more breaks and MVPA accumulated in durations of < 10 min vs. ≥10 min [14, 29].

While there was no statistically significant difference in mean accelerometer wear time between the baseline and 10-year follow-up assessments at the group level, within-subject differences in wear time were noted (mean difference = 1 min, but SD = 116 min). Since wear time is importantly associated with accelerometer-estimated durations of SB, LPA, and MVPA [30], we averaged baseline and follow-up wear time and rescaled all baseline and follow-up activity duration variables to this average. Ten-year differences in activity were calculated by subtracting rescaled baseline activity category duration from the rescaled follow-up activity category duration.

The short-form (SF)-12 was used to measure both physical and mental health status at baseline and 10-year follow-up. The SF-12 is an abbreviated version of the SF-36, developed as a general measure of health status for the Medical Outcomes Study [31]. The SF-12 was designed to provide a shorter questionnaire for large studies that yields mental component summary (MCS) and physical component summary (PCS) scores that are highly correlated to the SF-36 [32]. The SF-12 is commonly used to measure health status in longitudinal population studies [5, 33] and is associated with general health outcomes such as total medical expenditures [34]. Component scores range from 0 to 100, with a score of approximately 50 representing average mental or physical health status in middle-aged adults [33].

Height and weight were measured at baseline in light clothing and without shoes; body mass index (BMI) was calculated as kg/m^2^. Demographic characteristics including years of education, smoking (never, former, current), age, race, and sex were measured by standardized questionnaires at baseline.

#### Statistical methods

All analyses were conducted using Stata version 16 (STATA Corp, College Station, TX, USA). Baseline data were summarized across tertiles of baseline mean total activity, as well as tertiles of MCS and PCS, using means and standard deviations (SD) or numbers and percentages. Comparisons across tertiles used p-for-trend or chi-square tests, as appropriate. Though normality checks revealed baseline MVPA variables were not normally distributed, the original scale (min/day) was retained to aid in interpretation since results were similar with and without log transformation and model residuals using non-transformed MVPA were normally distributed and without influential points. Ten-year changes in MCS, PCS, and all activity variables were calculated and compared using paired *t* tests.

A series of linear regression models were fit to evaluate 10-year bidirectional associations between activity and MCS and PCS as described in detail below. Importantly, associations between the baseline value of the independent variable and 10-year changes in dependent variable establish temporality and are the primary association of interest in this bidirectional analysis. We also include and report associations between concurrent 10-year changes in the independent variable as a covariate; these 10-year-changes happen simultaneously, do not establish temporality, and were included to explore covariation in activity and self-rated health and to improve model fit.

##### Activity and 10-year changes in MCS and PCS

Initial models evaluated whether baseline and 10-year change in mean total activity was associated with 10-year changes in MCS and PCS. Next, to evaluate whether basic activity categories (i.e., SB, LPA, and MVPA) were associated with 10-year changes in MCS and PCS, a model was constructed including these activity categories and accelerometer wear time; one activity category (SB) was omitted as the reference category. Because durations in each intensity category are inter-related such that the total sum is equal to accelerometer wear time, this analysis examined associations when replacing SB with LPA and MVPA [35]. Lastly, the activity category analysis was repeated using expanded, short- and long-bout activity categories (i.e., long-bout SB, short-bout SB, LPA, short-bout MVPA, long-bout MVPA) with long-bout SB as the reference category. All beta coefficients were scaled to 1 SD of the independent variable (std. β) to aid in interpreting the meaningfulness of coefficients. All models were adjusted for baseline MCS or PCS, age, sex, education, smoking, study center, follow-up time, and average accelerometer wear time (Model 1). Model 2 added adjustment for baseline and 10-year change in BMI due the potential for BMI to be in the causal pathway between activity and physical and mental self-rated health [36].

##### PCS and MCS with 10-year changes in activity

To investigate the reverse direction of association, separate models evaluated whether baseline and 10-year change in MCS and PCS were associated with 10-year changes in mean total activity, SB, LPA, and MVPA as well short-bout and long-bout SB and MVPA. Again, std. β are reported (per 1 SD of the MCS or PCS variable) and models were adjusted for baseline activity, age, sex, education, smoking, study center, follow-up time, and average accelerometer wear time (Model 1) and then, additionally, by baseline and 10-year change in BMI (Model 2).

##### Race and sex interactions

The CARDIA Study was designed to study cardiovascular disease development across races (white, black) and sex (male, female). In this study, race and sex strata had sample sizes as follows: white men (*n* = 225); white women (*n* = 327); black men (*n* = 101); and black women (*n* = 241). Given these small subgroup samples, we conducted exploratory analyses evaluating race/sex interactions in the bidirectional associations between mean total activity, basic activity categories (SB, LPA, and MVPA), MCS, and PCS. As each analysis had at least one interaction term with *p* < 0.1 for both race and sex, we repeated analyses after stratification into race- and sex-specific groups (reported in Supplemental Tables).

## Results

Baseline participant characteristics are reported overall and by tertile of baseline mean total activity in Table 1. Participants were more often female, white, never smokers, had higher education degrees, and were classified, on average, as overweight. Though age, smoking status, and education did not differ by mean total activity tertile, participants in the higher tertiles had higher proportions of male and white participants as compared to the overall sample, and had lower BMI. Baseline MCS and PCS were slightly above the standardized population mean score of 50. Across higher tertiles of total activity, MCS did not differ while PCS was higher.

**Table 1:** Baseline participant characteristics (*n* = 894) overall and by tertiles of mean total activity

	Overall	Low Activity (118–297 cpm)	Moderate Activity (298–411 cpm)	High Activity (412–991 cpm)	*p*-value*
*Age, years*	45.1 (3.5)	45.1 (3.6)	45.1 (3.4)	45.1 (3.4)	0.834
*Sex*					
*Male*	326 (37%)	**93 (31%)**	**99 (34%)**	**134 (45%)**	**0.001**
*Female*	568 (63%)	**205 (69%)**	**199 (66%)**	**164 (55%)**	
*Race*					
*Black*	342 (38%)	**143 (48%)**	**115 (39%)**	**84 (28%)**	**< 0.001**
*White*	552 (62%)	**155 (52%)**	**183 (61%)**	**214 (72%)**	
*Smoking*					
*Never*	614 (69%)	207 (69%)	207 (69%)	200 (67%)	0.806
*Former*	169 (19%)	51 (17%)	56 (19%)	62 (21%)	
*Current*	111 (12%)	40 (13%)	35 (12%)	36 (12%)	
*Education*					
*≤ High School*	323 (36%)	113 (38%)	108 (36%)	102 (34%)	0.547
*Associate/Bachelor’s*	367 (41%)	123 (41%)	126 (42%)	118 (40%)	
*Postgraduate*	204 (23%)	62 (21%)	64 (21%)	78 (26%)	
*BMI, kg/m*^*2*^	28.5 (7.3)	**29.3 (6.8)**	**28.8 (6.8)**	**27.2 (8.1)**	**< 0.001**
*MCS, pts*	51.1 (8.8)	50.8 (9.1)	50.9 (9.3)	51.6 (8.1)	0.235
*PCS, pts*	52.2 (7.0)	**51.3 (7.0)**	**51.7 (7.6)**	**53.7 (6.2)**	**< 0.001**

**Statistical differences across tertiles are bolded.** Data are presented as mean (SD) or n (%)

*Abbreviations*: *BMI* body mass index, *cpm* counts per minute, *MCS* mental components scores, *PCS* physical component scores, *pts.* points

*Compared across tertiles using chi-square or linear test for trend

Some participant characteristics also differed across tertiles of MCS and PCS (Supplemental Table 1). Race (*p* = 0.005) and education (*p* = 0.018) categories differed across MCS tertiles; other characteristics were not associated with MCS. In the higher PCS tertiles, mean BMI was lower and there was a higher relative proportion of participants who were white, were never smokers, and had higher education (all *p* < 0.001).

### Associations of baseline and 10-year change in activity patterns with changes in self-rated health

Neither mean total activity nor basic activity categories were associated with 10-year changes in MCS (Table 2). In contrast, both higher baseline and 10-year increases in mean total activity were associated with favorable 10-year increases in PCS. Further, higher MVPA in place of lower SB (both baseline and 10-year changes) was associated with more favorable 10-year changes in PCS. Significant associations were slightly attenuated but remained statistically significant with adjustment for BMI.

**Table 2:** Association of baseline and 10-year changes in mean total activity and activity categories with changes in MCS and PCS (*n* = 894)

	MCS (pts)		PCS (pts)	
	*Model 1* *std. β (95% CI)*	*Model 2 (+BMI)* *std. β (95% CI)*	*Model 1* *std. β (95% CI)*	*Model 2 (+BMI)* *std. β (95% CI)*
***Mean Total Activity***				
*Baseline cpm*	0.30 (− 0.31, 0.91)	0.43 (− 0.19, 1.06)	**0.75 (0.23, 1.26)**	**0.53 (0.01, 1.05)**
*10-year change in cpm*	0.37 (− 0.23, 0.98)	0.46 (− 0.15, 1.07)	**1.12 (0.61, 1.62)**	**0.97 (0.47, 1.48)**
***Activity Categories***				
*SB*				
*Baseline & 10-year change*	ref.	ref.	ref.	ref.
*LPA*				
*Baseline*	−0.25 (− 0.89, 0.39)	− 0.20 (− 0.84, 0.44)	0.07 (− 0.46, 0.61)	−0.00 (− 0.53, 0.52)
*10-year change*	0.16 (− 0.45, 0.77)	0.22 (− 0.39, 0.83)	0.10 (− 0.41, 0.61)	0.01 (− 0.49, 0.51)
*MVPA*				
*Baseline*	0.49 (− 0.13, 1.12)	0.60 (− 0.03, 1.22)	**0.76 (0.24, 1.28)**	**0.60 (0.08, 1.12)**
*10-year change*	0.32 (−0.28, 0.91)	0.38 (−0.21, 0.97)	**1.17 (0.68, 1.66)**	**1.07 (0.58, 1.56)**

**Statistically significant results are bolded**

Standardized β are interpreted as the difference in MCS or PCS pts. per 1 SD difference in: total activity SD = 136 cpm; 10-year change in total activity SD = 139 cpm; LPA SD = 85 min per day; 10-year change in LPA SD = 88 min per day; MVPA SD = 23 min per day; 10-year change in MVPA SD = 23 min per day

Model 1 includes LPA and MVPA in the activity category analysis; both analyses also adjust for baseline value of MCS or PCS, follow-up time, age, race, gender, education, smoking, center, and average accelerometer wear time

Model 2 adds adjustment for baseline and 10-year change in BMI

*Abbreviations*: *BMI* body mass index, *cpm* counts per minute, *LPA* light-intensity physical activity, *MCS* mental component score, *MVPA* moderate-to-vigorous intensity physical activity, *PCS* physical component score, *pts.* points, *ref.* reference category, *SB* stationary behavior

Table 3 reports associations between baseline and 10-year changes in expanded activity categories (with long bout SB as the reference category) with 10-year changes in MCS and PCS. Only higher baseline short-bout MVPA was significantly associated with a more favorable 10-year change in MCS. Higher baseline long-bout MVPA as well 10-year changes in both short- and long-bout MVPA, with correspondingly lower levels of long-bout SB (reference category), were associated with 10-year increases in PCS.

**Table 3:** Association of baseline and 10-year Changes in SB (short and long bouts), LPA, and MVPA (short and long bout) and changes in MCS and PCS (*n* = 894)

	MCS (pts)		PCS (pts)	
	*Model 1* *std. β (95% CI)*	*Model 2 (+BMI)* *std. β (95% CI)*	*Model 1* *std. β (95% CI)*	*Model 2 (+BMI)* *std. β (95% CI)*
*SB (long-bout)*				
*Baseline & 10-year change*	ref.	ref.	ref.	ref.
*SB (short-bout)*				
*Baseline*	0.61 (− 0.33, 1.55)	0.58 (− 0.36, 1.52)	0.03 (− 0.75, 0.81)	0.08 (− 0.69, 0.86)
*10-year change*	0.70 (− 0.01, 1.40)	0.66 (− 0.05, 1.36)	−0.22 (− 0.80, 0.36)	−0.15 (− 0.72, 0.43)
*LPA*				
*Baseline*	−0.40 (− 1.18, 0.38)	− 0.32 (− 1.11, 0.46)	0.20 (− 0.44, 0.85)	0.07 (− 0.57, 0.72)
*10-year change*	0.20 (− 0.48, 0.88)	0.25 (− 0.43, 0.93)	−0.09 (− 0.65, 0.48)	−0.18 (− 0.73, 0.38)
*MVPA (short-bout)*				
*Baseline*	**0.92 (0.14, 1.71)**	**0.89 (0.10, 1.67)**	0.30 (−0.35, 0.95)	0.37 (−0.27, 1.02)
*10-year change*	0.47 (−0.28, 1.22)	0.48 (−0.27, 1.23)	**0.99 (0.37, 1.61)**	**0.98 (0.37, 1.60)**
*MVPA (long-bout)*				
*Baseline*	0.50 (−0.47, 1.46)	0.65 (−0.33, 1.62)	**1.47 (0.66, 2.28)**	**1.21 (0.40, 2.01)**
*10-year change*	0.51 (−0.42, 1.43)	0.58 (−0.35, 1.50)	**1.26 (0.49, 2.02)**	**1.13 (0.37, 1.89)**

**Statistically significant results are bolded**

Standardized β are interpreted as the difference in MCS or PCS pts. per 1 SD difference in: SB (short) SD = 88 min per day; 10-year change in SB (short) = 75 min per day; LPA SD = 85 min per day; 10-year change in LPA SD = 111 min per day; MVPA (short) SD = 13 min per day; 10-year change in MVPA (short) SD = 13 min per day; MVPA (long) SD = 18 min per day; 10-year change in MVPA (long) = 19 min per day

*Model 1 includes all activity variables and also adjusts for baseline value of MCS or PCS, follow-up time, accelerometer wear time, age, race, gender, education, smoking, and center

**Model 2 adds adjustment for baseline and 10-year change in BMI

*Abbreviations*: *BMI* body mass index, *cpm* counts per minute, *LPA* light-intensity physical activity, *MCS* mental component score, *MVPA* moderate-to-vigorous intensity physical activity, *PCS* physical component score, *pts.* points, *ref.* reference category, *SB* stationary behavior

### Associations of baseline and 10-year change in self-rated health with changes in activity patterns

Higher baseline MCS was associated with more positive 10-year changes in mean total activity and was associated with greater decreases in SB over the 10-year follow-up in fully adjusted models (Model 2, Table 4). Ten-year change in MCS was not associated with any activity categories. Both higher baseline PCS and greater 10-year changes in PCS were each associated with favorable 10-year changes in total activity, SB (only in Model 1), and MVPA.

**Table 4:** Association of baseline and changes in MCS and PCS on mean total activity, SB, LPA, and MVPA (*n* = 894)

	Mean Total Activity (cpm)		SB (min/day)		LPA (min/day)		MVPA (min/day)	
	*Model 1*	*Model 2* *(+BMI)*	*Model 1*	*Model 2* *(+BMI)*	*Model 1*	*Model 2 (+BMI)*	*Model 1*	*Model 2 (+BMI)*
	*std. β (95% CI)*		*std. β (95% CI)*		*std. β (95% CI)*		*std. (95% CI)*	
**MCS**								
*Baseline*	8.9 (−0.5, 18.2)	**9.8 (0.5, 19.1)**	−6.2 (− 12.8, 0.3)	**− 6.6 (− 13.1, − 0.1**)	4.8 (− 1.1, 10.8)	5.1 (− 0.9, 11.1)	1.3 (− 0.3, 2.9)	1.4 (− 0.2, 3.1)
*10-yr change*	5.6 (− 3.5, 14.7)	6.9 (− 2.2, 15.9)	− 3.5 (− 9.9, 2.9)	−3.9 (− 10.2, 2.4)	2.4 (− 3.5, 8.3)	2.7 (− 3.1, 8.6)	1.0 (− 0.6, 2.6)	1.2 (− 0.4, 2.8)
**PCS**								
*Baseline*	**18.4 (9.2, 27.2)**	**15.7 (6.3, 25.0)**	**−9.0 (−15.2, 2.7)**	− 6.6 (− 13.1, 0.0)	5.8 (− 0.1, 11.8)	3.8 (− 2.4, 10.0)	**3.2 (1.6, 4.7)**	**2.8 (1.2, 4.5)**
*10-yr change*	**19.1 (10.5, 27.7)**	**16.9 (8.1, 25.6**)	**−7.6 (− 13.7, − 1.5)**	**−6.5 (− 12.6, − 0.3)**	3.10 (− 2.6, 8.8)	2.1 (− 3.6, 7.9)	**3.7 (2.2, 5.2)**	**3.5 (1.9, 5.0)**

**Statistically significant results are bolded**

Model 1 is adjusted for baseline value of the activity variable, follow-up time, average accelerometer wear time, age, race, gender, education, smoking, and center

Model 2 adds adjustment for baseline and 10-year change in BMI

MCS SD = 8.83 points; MCS 10-year change SD = 9.07 points; PCS SD = 7.00 points; PCS 10-year change SD = 7.30 point

*Abbreviations*: *BMI* body mass index, *cpm* counts per minute, *LPA* light-intensity physical activity, *MCS* mental component score, *MVPA* moderate-to-vigorous intensity physical activity, *PCS* physical component score, *pts.* points, *ref.* reference category, *SB* stationary behavior

When evaluating associations of self-rated health on 10-year changes in extended activity categories (short−/long-bout SB and MVPA), higher baseline MCS was inversely associated with changes in long-bout SB and directly associated with changes in short-bout SB and short-bout MVPA (Table 5). Greater baseline and 10-year changes in PCS were each inversely associated changes in short-bout SB and directly associated with changes in short- and long-bout MVPA.

**Table 5:** Association of baseline and changes in MCS and PCS on long-bout and short-bout SB and MVPA (*n* = 894)

	Long-bout SB (min/day)		Short-bout SB (min/day)		Short-bout MVPA (min/day)		Long-bout MVPA (min/day)	
	*Model 1*	*Model 2* *(+BMI)*	*Model 1*	*Model 2* *(+BMI)*	*Model 1*	*Model 2 (+BMI)*	*Model 1*	*Model 2 (+BMI)*
	*std. β (95% CI)*		*std. β (95% CI)*		*std. β (95% CI)*		*std. β (95% CI)*	
**MCS**								
*Baseline*	−**13.3 (− 20.1, − 6.6)**	**−13.6 (− 20.3, − 7.8)**	**6.6 (2.2, 11.0)**	**6.3 (1.9, 10.7)**	**0.8 (0.0, 1.6)**	**0.9 (0.1, 1.7)**	0.7 (−0.6, 2.1)	0.8 (− 0.6, 2.2)
*10-yr change*	−5.9 (− 12.5, 0.7)	− 6.3 (− 12.9, 0.3)	2.7 (− 1.6, 7.0)	2.2 (− 2.1, 6.5)	0.5 (− 0.3, 1.3)	0.5 (− 0.2, 1.3)	0.7 (− 0.7, 2.0)	0.8 (− 0.5, 2.1)
**PCS**								
*Baseline*	−4.0 (−10.6, 2.6)	− 2.6 (− 9.6, 4.3)	**−5.1 (− 9.4, − 0.9)**	− 4.0 (− 8.4, 0.5)	**1.3 (0.6, 2.1)**	**1.2 (0.4, 2.0)**	**1.9 (0.6, 3.2)**	**1.7 (0.4, 3.1)**
*10-yr change*	−2.8 (− 9.2, 3.5)	−2.0 (− 8.5, 4.5)	**−4.6 (− 8.7, − 0.5)**	−3.7 (− 7.9, 0.5)	**1.5 (0.7, 2.2)**	**1.4 (0.6, 2.2)**	**2.2 (1.0, 3.5)**	**2.1 (0.8, 3.4)**

**Statistically significant results are bolded**

Model 1 is adjusted for baseline value of the activity variable, follow-up time, average accelerometer wear time, age, race, gender, education, smoking, and center

Model 2 adds adjustment for baseline and 10-year change in BMI

MCS SD = 8.83 points; MCS 10-year change SD = 9.07 points; PCS SD = 7.00 points; PCS 10-year change SD = 7.30 point

*Abbreviations*: *BMI* body mass index, *cpm* counts per minute, *LPA* light-intensity physical activity, *MCS* mental component score, *MVPA* moderate-to-vigorous intensity physical activity, *PCS* physical component score, *pts.* points, *ref.* reference category, *SB* stationary behavior

### Associations after stratification by race and sex

Bidirectional associations after stratification by race and sex are reported in Supplemental Tables 2 and 3. Baseline activity was not associated with 10-year changes in self-rated health within race/sex groups; yet, baseline self-rated health (especially PCS) was associated with unfavorable activity level changes in all groups except white women.

## Discussion

Among 894 white and black men and women during midlife, we investigated bidirectional associations of accelerometer-measured SB and activity with self-rated mental and physical health. Longitudinal associations in each direction, between baseline values of the independent variable and 10-year changes in the dependent variable, are summarized in the Figure. Overall, higher baseline MVPA was most consistently associated with preserving self-rated physical health and, to a lesser extent, self-rated mental health. Higher baseline LPA (in exchange for SB) was not associated with preservation of either domain of self-rated health. During the same interval, higher baseline self-rated mental and physical health were both associated with more favorable SB and MVPA at the 10-year follow-up.

**Fig. 1:**
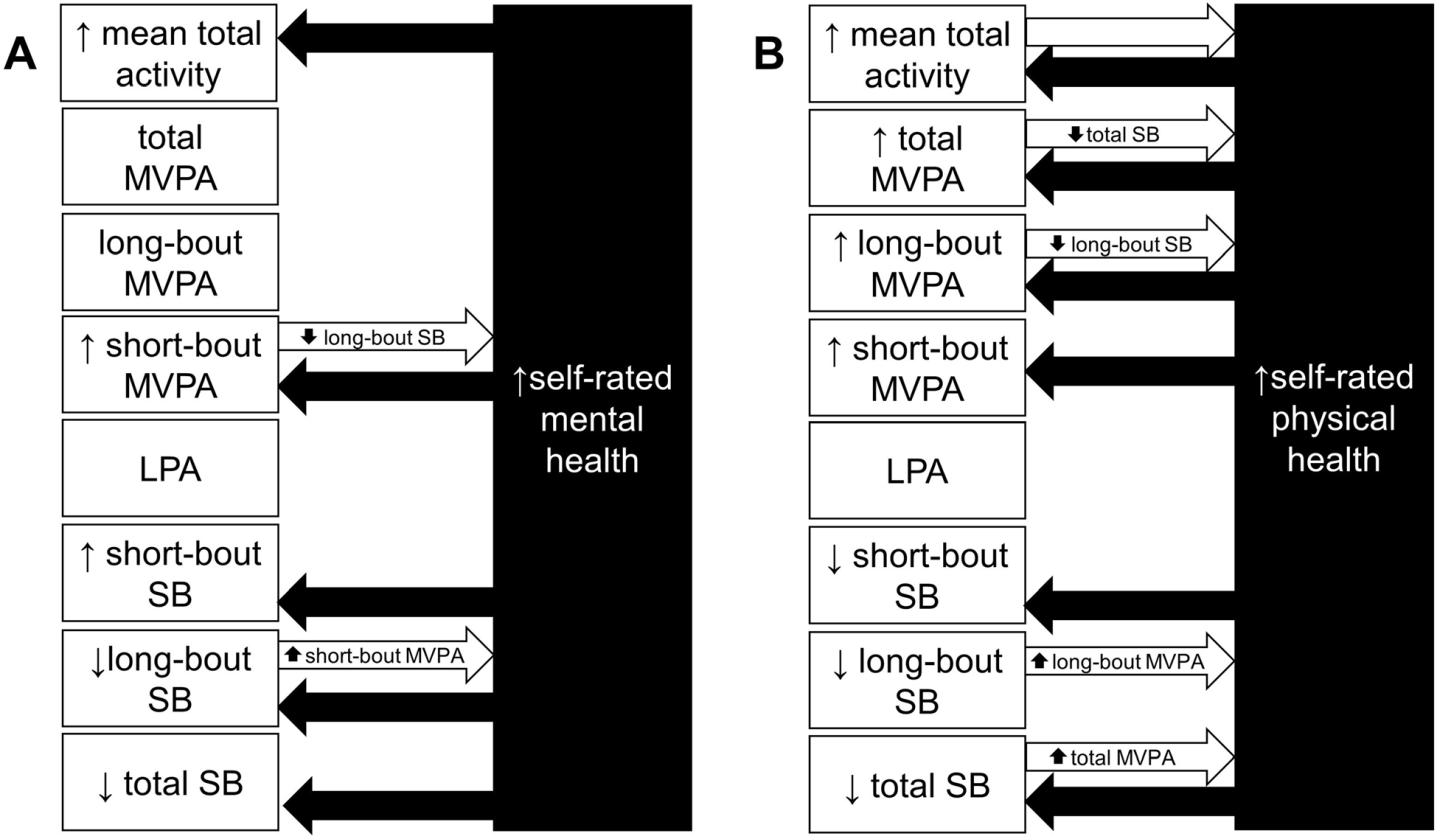
Summary of Bidirectional Associations between Accelerometer-Measured Activity Levels and Self-rated Mental Health (**A**) and Self-rated Physical Health (**B**). Associations between baseline activity levels and 10-year changes in self-rated health are represented by white arrows (including the reference group, where appropriate); associations of baseline self-rated health and 10-year changes in activity levels are represented by the black arrows

Limited associations were observed between baseline activity patterns and 10-year changes in self-rated mental health (Fig. 1a, white arrows). Only higher levels of baseline short-bout MVPA (replacing long-bout SB) were associated with greater 10-year increases in MCS. In contrast, associations of self-rated physical health and 10-year changes in activity levels were more apparent (Fig. 1b). Higher baseline mean total activity and MVPA (both total and long-bout MVPA) were associated with more favorable 10-year changes in PCS. These associations were independent of significant associations also observed between 10-year changes in mean total activity/MVPA with 10-year changes in PCS (not represented in the Figure). Taken together, these data suggest that both short-bout MVPA (for MCS) and long-bout MVPA (for PCS) could help preserve self-rated health during midlife. Importantly, the magnitude of associations between baseline activity (per SD) and self-rated health outcomes were small, about 1 to 1.5 pts. in self-rated health. However, these effect sizes were similar to the observed 10-year changes in MCS (+ 1.05 pts) and PCS (− 1.54 pts), and therefore may be clinically important.

More associations were observed in the reverse direction (Fig. 1a). Higher baseline MCS was directly associated with 10-year change in mean total activity, reflecting relative decreases in total and long-bout SB and increases in short-bout SB and short-bout MVPA. Higher baseline PCS was also associated with more favorable 10-year changes in activity levels (Fig. 1b) including greater 10-year changes in mean total activity and MVPA (total, short, and long-bout) and decreases in SB (total and short-bout). As above, associations were small in magnitude. For example, per SD of baseline MCS, 10-year change in long-bout SB was 13.57 min lower per day. Ten-year change in MVPA was about 3 min/day higher per SD of baseline PCS, which would translate to ~ 20 min per week. Again, however, when considering that concurrent 10-year changes in this cohort were an unfavorable + 41 min/day of long-bout SB and − 6 min/day of MVPA, higher baseline MCS and PCS could attenuate a meaningful proportion of these longitudinal activity changes across midlife.

Our findings generally agree with the established direct association between higher MVPA and higher self-reported health and/or quality of life. Reviews and meta-analyses of observational research as well as exercise interventions demonstrate a positive effect of MVPA on various measures of self-reported health across a range of patient populations [11–13, 37]. Associations on whole-day activity patterns or components of physical activity (SB, LPA, and MVPA) and self-reported health have been less studied, especially in younger or middle-aged adults. In a systematic review of correlates of sedentary behavior in adults [38], the majority of studies reported an inverse association between total sitting time and MCS (3 of 5), while most studies found null associations between total sitting time and PCS (4 of 6). However, the authors noted important limitations to the studies informing the systematic review in that almost all used self-reported measures of sedentary behavior and cross-sectional designs. One cross-sectional study evaluated whether total and mental self-rated health were associated with accelerometer-measured activity among 921 adults in the Health Survey for England. General self-rated health, but not mental self-rated health, was directly associated with MVPA and no associations were observed between self-rated health and SB or LPA [39]. In contrast, a cross-sectional study from South Korea found that isotemporal reallocation of time to higher intensity accelerometer-measured activity (i.e., SB to LPA or MVPA; LPA to MVPA) was associated with better overall self-rated health among young and older adults, but not middle-aged adults [40]. Finally, and most similar to our study, a recent cross-sectional, compositional data analysis among 430 young and middle-aged adults in Australia identified that allocations with higher accelerometer-measured MVPA (with lower SB, LPA, or sleep) were consistently associated with higher PCS, but not MCS, from the SF-12. Also, similar to our observations, compositions with less SB and more LPA were not associated with better self-rated health, and were in fact associated with worse PCS [36]. Our longitudinal study extends these largely cross-sectional findings and sheds more light on temporal associations, where more baseline MVPA in particular may help preserve mental and physical self-rated health across midlife.

We are unaware of other research evaluating longitudinal associations of baseline self-rated health and prospective changes in activity patterns. However, the associations we observed, where higher self-rated health was associated with more favorable 10-year changes in activity levels, are consistent with our *a priori* hypotheses. This is based on established research that identifies lower perceived mental/physical health, mental health symptoms and disorders, and reduced physical function as barriers to physical activity and/or adherence in exercise interventions [16, 18] Our longitudinal findings suggest that these barriers may have a progressive impact on activity patterns over time, identifying that subgroups with lower self-rated mental and physical health may be at higher risk for declining activity levels. Moreover, mental and physical self-rated health may additionally need to be addressed to improve overall activity levels in clinical and community settings.

This analysis is strengthened by repeated, accelerometer-based measures of SB and activity and self-rated health in a large, biracial cohort. We were able to adjust for important covariates, including progressive adjustment for BMI which, though a potential mediator of associations between activity and self-rated health, did not typically explain observed associations. However, external validity could be limited by differences in our analytic sample that only included participants with complete accelerometry and SF-12 data as compared to all the CARDIA participants who completed the follow-up exams of interest. Specifically, our sample tended to be healthier (lower BMI and higher PCS) and to have a higher proportion of whites and females. Though uncertain, we expect this could have weakened the observed bidirectional associations. Also, we were able to associate changes in activity and self-rated health over only two timepoints; more frequent repeated measures for example using ecological momentary assessment could strengthen conclusions. Our purposeful classification of time spent in activity with < 100 cpm as ‘stationary’ reflects the limited ability of the devices used to detect posture as well as non-ambulatory movement. Though stationary time is often used as an estimation of sedentary time, it also includes other behaviors (e.g., stationary standing) and should be interpreted with this caveat in mind [16]. Also, though we carefully chose cut points for short- and long-bout SB and MVPA based on the previous literature [2, 14, 27–29], these remain somewhat arbitrary and future dose-response analyses are warranted. Further, we lacked contextual information about the activity behaviors we measured, and associations could have differed by domain (e.g., occupational vs. leisure activities) [41]. Lastly, despite the strengths of SF-12 to measure overall well-being with strong predictive ability for health outcomes, individual personality or perceptions (e.g., optimism, stoicism, or hypochondria) may have introduced error in this self-reported measure [5, 34].

## Conclusions

Higher MVPA in exchange for lower SB in adults at midlife was associated with improved preservation of mental and physical self-rated health. At the same time, lower self-rated health was associated with unfavorable longitudinal shifts toward more SB and less MVPA. These co-occurring effects have implications for research and practice. First, cross-sectional associations between activity levels and self-rated health are likely overestimating effects in any one direction. Second, poor activity levels (i.e., high SB and low MVPA) and low self-rated health likely result in a feedback loop of adverse bidirectional effects. This phenomenon would suggest that individuals with poor self-rated health and activity levels are at high risk for further declines. Health promotion programming and interventions might need to address self-rated health in additional to low levels of activity to more effectively preserve or improve health during midlife.

## Supplementary Information

**Additional file 1: Supplemental Table 1**. Baseline Participant Characteristics (*n*=894) by Tertiles of MCS and PCS. **Supplemental Table 2.** Baseline and 10-year Follow-up Activity Patterns in CARDIA. Association of Baseline and Changes in Total Activity and Simple Activity Categories with Changes in MCS and PCS, Race/Sex Stratified Results. **Supplemental Table 3**. Association (std. β) of Baseline and Changes in MCS and PCS on Mean Total Activity, SB, LPA, and MVPA, Race/Sex Stratified Results.

**Additional file 2: Supplemental Figure 1.** Baseline and 10-year Follow-up Self-rated Health in CARDIA.

## Data Availability

The data that support the findings of this study are available from the CARDIA Study coordinating center upon reasonable request (https://www.cardia.dopm.uab.edu/).

## Abbreviations

LPA: Light intensity physical activity
MCS: Mental component score
MVPA: Moderate-to-vigorous intensity physical activity
PCS: Physical component score
QoL: Quality of life
SB: Stationary behavior
SD: Standard deviation
SF-12: Short-form 12

## References

[1] Piercy KL, Troiano RP, Ballard RM, Carlson SA, Fulton JE, Galuska DA, George SM, Olson RD (2018). The physical activity guidelines for Americans. JAMA.

[2] Diaz KM, Howard VJ, Hutto B, Colabianchi N, Vena JE, Safford MM, Blair SN, Hooker SP (2017). Patterns of sedentary behavior and mortality in US middle-aged and older adults: a national cohort study. Ann Intern Med.

[3] Katzmarzyk PT, Powell KE, Jakicic JM, Troiano RP, Piercy K, Tennant B (2019). Sedentary behavior and health: update from the 2018 physical activity guidelines advisory committee. Med Sci Sports Exerc.

[4] Crimmins EM, Beltrán-Sánchez H (2010). Mortality and morbidity trends: is there compression of morbidity?. J Gerontol Series B.

[5] Jenkinson C, Layte R, Jenkinson D, Lawrence K, Petersen S, Paice C, Stradling J (1997). A shorter form health survey: can the SF-12 replicate results from the SF-36 in longitudinal studies?. J Public Health.

[6] Erickson KI, Hillman C, Stillman CM, Ballard RM, Bloodgood B, Conroy DE, Macko R, Marquez DX, Petruzzello SJ, Powell KE, FOR 2018 PHYSICAL ACTIVITY GUIDELINES ADVISORY COMMITTEE* (2019). Physical activity, cognition, and brain outcomes: a review of the 2018 physical activity guidelines. Med Sci Sports Exerc.

[7] Huang Y, Li L, Gan Y, Wang C, Jiang H, Cao S, Lu Z (2020). Sedentary behaviors and risk of depression: a meta-analysis of prospective studies. Transl Psychiatry.

[8] Rebar AL, Stanton R, Geard D, Short C, Duncan MJ, Vandelanotte C (2015). A meta-meta-analysis of the effect of physical activity on depression and anxiety in non-clinical adult populations. Health Psychol Rev.

[9] Powell KE, King AC, Buchner DM, Campbell WW, DiPietro L, Erickson KI, Hillman CH, Jakicic JM, Janz KF, Katzmarzyk PT, Kraus WE, Macko RF, Marquez DX, McTiernan A, Pate RR, Pescatello LS, Whitt-Glover MC (2019). The scientific Foundation for the Physical Activity. J Phys Act Health.

[10] Lavie CJ, Ozemek C, Carbone S, Katzmarzyk PT, Blair SN (2019). Sedentary behavior, exercise, and cardiovascular health. Circ Res.

[11] Rejeski WJ, Brawley LR, Shumaker SA (1996). Physical activity and health-related quality of life. Exerc Sport Sci Rev.

[12] Conn VS, Hafdahl AR, Brown LM (2009). Meta-analysis of quality-of-life outcomes from physical activity interventions. Nurs Res.

[13] Bize R, Johnson JA, Plotnikoff RC (2007). Physical activity level and health-related quality of life in the general adult population: a systematic review. Prev Med.

[14] JAKICIC JM, KRAUS WE, POWELL KE, CAMPBELL WW, JANZ KF, TROIANO RP (2019). Association between bout duration of physical activity and health: systematic review. Med Sci Sports Exerc.

[15] Molarius A, Janson S (2002). Self-rated health, chronic diseases, and symptoms among middle-aged and elderly men and women. J Clin Epidemiol.

[16] Tremblay MS, Aubert S, Barnes JD, Saunders TJ, Carson V, Latimer-Cheung AE (2017). Sedentary behavior research network (SBRN) - terminology consensus project process and outcome. Int J Behav Nutr Phys Act.

[17] Barone Gibbs B, Aaby D, Siddique J, Reis JP, Sternfeld B, Whitaker K, Pettee Gabriel K (2020). Bidirectional 10-year associations of accelerometer-measured sedentary behavior and activity categories with weight among middle-aged adults. Int J Obes.

[18] DiPietro L (2001). Physical activity in aging: changes in patterns and their relationship to health and function. J Gerontol A Biol Sci Med Sci.

[19] King AC (2001). Interventions to promote physical activity by older adults. J Gerontol A Biol Sci Med Sci.

[20] Friedman GD, Cutter GR, Donahue RP, Hughes GH, Hulley SB, Jacobs DR, Liu K, Savage PJ (1988). CARDIA: study design, recruitment, and some characteristics of the examined subjects. J Clin Epidemiol.

[21] Pettee Gabriel K, Sidney S, Jacobs DR, Whitaker KM, Carnethon MR, Lewis CE, Schreiner PJ, Malkani RI, Shikany JM, Reis JP, Sternfeld B (2018). Ten-year changes in accelerometer-based physical activity and sedentary time during midlife: the CARDIA study. Am J Epidemiol.

[22] Whitaker KM, Pettee Gabriel K, Jacobs DR, Sidney S, Sternfeld B (2018). Comparison of two generations of ActiGraph accelerometers: the CARDIA study. Med Sci Sports Exerc.

[23] Tudor-Locke C. A catalog of rules, variables, and definitions applied to accelerometer data in the National Health and nutrition examination survey, 2003–2006. Prev Chronic Dis. 2012;9. 10.5888/pcd9.110332 PMC3457743 22698174

[24] Troiano RP, Berrigan D, Dodd KW, Masse LC, Tilert T, McDowell M (2008). Physical activity in the United States measured by accelerometer. Med Sci Sports Exerc.

[25] Masse LC, Fuemmeler BF, Anderson CB, Matthews CE, Trost SG, Catellier DJ (2005). Accelerometer data reduction: a comparison of four reduction algorithms on select outcome variables. Med Sci Sports Exerc.

[26] Freedson P, Bowles HR, Troiano R, Haskell W (2012). Assessment of physical activity using wearable monitors: recommendations for monitor calibration and use in the field. Med Sci Sports Exerc.

[27] Kim Y, Welk GJ, Braun SI, Kang M (2015). Extracting objective estimates of sedentary behavior from accelerometer data: measurement considerations for surveillance and research applications. PLoS One.

[28] Dunstan DW, Howard B, Healy GN, Owen N (2012). Too much sitting--a health hazard. Diabetes Res Clin Pract.

[29] 2018 Physical Activity Guidelines Advisory Committee (2018). 2018 Physical Activity Guidelines Advisory Committee Scientific Report.

[30] Healy GN, Clark BK, Winkler EAH, Gardiner PA, Brown WJ, Matthews CE (2011). Measurement of Adults' sedentary time in population-based studies. Am J Prev Med.

[31] Ware JE, Sherbourne CD (1992). The MOS 36-item short-form health survey (SF-36). I. Conceptual framework and item selection. Med Care.

[32] Ware JE, Kosinski M, Keller SD (1996). A 12-item Short-form health survey: construction of scales and preliminary tests of reliability and validity. Med Care.

[33] Hanmer J, Lawrence WF, Anderson JP, Kaplan RM, Fryback DG (2006). Report of nationally representative values for the noninstitutionalized US adult population for 7 health-related quality-of-life scores. Med Decis Mak.

[34] Fleishman JA, Cohen JW, Manning WG, Kosinski M (2006). Using the SF-12 health status measure to improve predictions of medical expenditures. Med Care.

[35] Mekary RA, Willett WC, Hu FB, Ding EL (2009). Isotemporal substitution paradigm for physical activity epidemiology and weight change. Am J Epidemiol.

[36] Rachel GC, Dorothea D, Timothy O, Ronald P, Corneel V, Jillian R (2020). The association between time-use behaviors and physical and mental well-being in adults: a compositional Isotemporal substitution analysis. J Phys Act Health.

[37] Rabel M, Meisinger C, Peters A, Holle R, Laxy M (2017). The longitudinal association between change in physical activity, weight, and health-related quality of life: results from the population-based KORA S4/F4/FF4 cohort study. PLoS One.

[38] Prince SA, Reed JL, McFetridge C, Tremblay MS, Reid RD (2017). Correlates of sedentary behaviour in adults: a systematic review. Obes Rev.

[39] Hamer M, Stamatakis E (2010). Objectively assessed physical activity, fitness and subjective wellbeing. Ment Health Phys Act.

[40] Park S, Park S-Y, Oh G, Yoon EJ, Oh I-H (2020). Association between reallocation behaviors and subjective health and stress in south Korean adults: an Isotemporal substitution model. Int J Environ Res Public Health.

[41] Holtermann A, Krause N, van der Beek AJ, Straker L (2018). The physical activity paradox: six reasons why occupational physical activity (OPA) does not confer the cardiovascular health benefits that leisure time physical activity does. Br J Sports Med.

