# Supplementary material for: Bidirectional associations of accelerometer-derived physical activity and stationary behavior with self-reported mental and physical health during midlife
Source: Int J Behav Nutr Phys Act. 2021 Jun 6;18:74. doi: 10.1186/s12966-021-01145-4 (PMC8180096; doi:10.1186/s12966-021-01145-4)
Supplement: Supplementary file 1 — Additional file 1: Supplemental Table 1. Baseline Participant Characteristics (n=894) by Tertiles of MCS and PCS. Supplemental Table 2. Baseline and 10-year Follow-up Activity Patterns in CARDIA. Association of Baseline and Changes in Total Activity and Simple Activity Categories with Changes in MCS and PCS, Race/Sex Stratified Results. Supplemental Table 3. Association (std. β) of Baseline and Changes in MCS and PCS on Mean Total Activity, SB, LPA, and MVPA, Race/Sex Stratified Results. [file 12966_2021_1145_MOESM1_ESM.docx]

**Supplemental Table 1. Baseline Participant Characteristics (n=894) by Tertiles of MCS and PCS**

|  | Low MCS  (17-50 pts) | Moderate MCS  (51-55 pts) | High MCS  (56-69 pts) | p-value |
| --- | --- | --- | --- | --- |
| *Age, years* | 44.8 (3.4) | 45.4 (3.4) | 45.1 (3.6) | 0.218 |
| *Sex*  *Male*  *Female* | 101 (34%)  197 (66%) | 108 (36%)  191 (64%) | 117 (39%)  180 (61%) | 0.374 |
| *Race*  *Black*  *White* | **125 (42%)**  **173 (58%)** | **92 (31%)**  **207 (69%)** | **125 (42%)**  **172 (58%)** | **0.005** |
| *Smoking*  *Never*  *Former*  *Current* | 188 (63%)  64 (21%)  46 (15%) | 218 (73%)  50 (17%)  31 (10%) | 208 (70%)  55 (19%)  34 (11%) | 0.114 |
| *Education*  *≤ High School*  *Associate/Bachelor’s*  *Postgraduate* | **127 (43%)**  **105 (35%)**  **66 (22%)** | **99 (33%)**  **123 (41%)**  **77 (26%)** | **97 (33%)**  **139 (47%)**  **61 (21%)** | **0.018** |
| *BMI, kg/m^2^* | 28.5 (7.0) | 27.5 (5.6) | 29.3 (8.9) | 0.200 |
|  |  |  |  |  |
|  | **Low PCS**  **(17-52 pts)** | **Moderate PCS**  **(53-55 pts)** | **High PCS**  **(56-64 pts)** | **p-value** |
| *Age, years* | 45.2 (3.5) | 44.9 (3.5) | 45.2 (3.5) | 0.992 |
| *Sex*  *Male*  *Female* | 98 (33%)  203 (67%) | 124 (41%)  179 (59%) | 104 (36%)  186 (64%) | 0.099 |
| *Race*  *Black*  *White* | **139 (46%)**  **162 (54%)** | **122 (40%)**  **181 (60%)** | **81 (28%)**  **209 (72%)** | **<0.001** |
| *Smoking*  *Never*  *Former*  *Current* | **178 (59%)**  **62 (21%)**  **61 (20%)** | **219 (72%)**  **52 (17%)**  **32 (11%)** | **217 (75%)**  **55 (19%)**  **18 (6%)** | **<0.001** |
| *Education*  *≤ High School*  *Associate/Bachelor’s*  *Postgraduate* | **136 (45%)**  **121 (40%)**  **44 (15%)** | **110 (36%)**  **127 (42%)**  **66 (22%)** | **77 (27%)**  **119 (41%)**  **94 (32%)** | **<0.001** |
| *BMI, kg/m^2^* | **30.6 (7.5)** | **28.9 (8.4)** | **25.8 (4.5)** | **<0.001** |

*Compared across tertiles using chi-square or linear test for trend

**Statistically significant differences by tertile are bolded.** Data are presented as mean (SD) or n (%)

Abbreviations: BMI, body mass index; cpm, counts per minute; MCS, mental component score; PCS, physical component score; pts, points;

**Supplemental Table 2. Baseline and 10-year Follow-up Activity Patterns in CARDIA**

| 1. CPM   Baseline: 370 (136) cpm  Follow-up: 318 (140) cpm  Change: -52 (139) cpm (p<0.001) | 1. SB   Baseline: 500 (104) min/day  Follow-up: 539 (108) min/day  Change: +38 (96) min/day (p<0.001) | 1. SB (long-bout)   Baseline: 111 (72) min/day  Follow-up: 153 (96) min/day  Change: +41 (95) min/day (p<0.001) | 1. SB (short-bout)   Baseline: 389 (68) min/day  Follow-up: 386 (69) min/day  Change: -4 (71) min/day (p=0.132) |
| --- | --- | --- | --- |

| 1. LPA   Baseline: 360 (85) min/day  Follow-up: 328 (90) min/day  Change: -32 (88) min/day (p<0.001) | 1. MVPA   Baseline: 36 (23) min/day  Follow-up: 29 (26) min/day  Change: -6 (23) min/day (p<0.001) | 1. MVPA (short-bout)   Baseline: 21 (13) min/day  Follow-up: 15 (12) min/day  Change: -6 (13) min/day (p<0.001) | 1. MVPA (long-bout)   Baseline: 15 (18) min/day  Follow-up: 15 (21) min/day  Change: 0 (19) min/day (p=0.950) |
| --- | --- | --- | --- |

Data presented as mean (SD)

**Supplemental Table 2. Association of Baseline and Changes in Total Activity and Simple Activity Categories with Changes in MCS and PCS, Race/Sex Stratified Results**

|  |  | **MCS (pts)** | | | |  | **PCS (pts)** | | | |
| --- | --- | --- | --- | --- | --- | --- | --- | --- | --- | --- |
|  |  | *White Men*  *(n=225)*  std. β | *Black Men*  *(n=101)*  std. β | *White Women*  *(n=327)*  std. β | *Black Women*  *(n=241)*  std. β |  | *White Men*  *(n=225)*  std. β | *Black Men*  *(n=101)*  std. β | *White Women*  *(n=327)*  std. β | *Black Women*  *(n=241)*  std. β |
| **Mean Total Activity** |  |  |  |  |  |  |  |  |  |  |
| *Baseline cpm* |  | 0.78 | -0.59 | -0.05 | 0.35 |  | 0.56 | 1.51 | 0.76 | 0.54 |
| *10-year change in cpm* |  | **1.98***** | 0.04 | -0.18 | -0.61 |  | 0.84 | **2.25*** | 0.53 | **2.00***** |
|  |  |  |  |  |  |  |  |  |  |  |
| **Activity Categories** |  |  |  |  |  |  |  |  |  |  |
| *SB*  *Baseline & 10-year*  *change* |  | ref. | ref. | ref. | ref. |  | ref. | ref. | ref. | ref. |
| *LPA*  *Baseline*  *10-year change* |  | -0.28  0.76 | -1.65  -0.57 | -0.26  0.20 | -0.07  -0.57 |  | 0.07  -0.18 | -0.75  1.33 | 0.27  -0.43 | 0.33  0.70 |
| *MVPA*  *Baseline*  *10-year change* |  | 0.75  **1.45**** | 0.20  -0.01 | 0.12  -0.37 | 0.93  0.53 |  | 0.50  **0.94*** | 1.82  1.39 | 0.71  **0.90*** | 0.41  **1.66**** |

**Statistically significant results are bolded.**

Standardized β are interpreted as the difference in MCS or PCS per 1 SD difference in activity metric. Standardized β are adjusted for LPA and MVPA in the activity category analysis; both analyses also adjust for baseline value of MCS or PCS, follow-up time, age, education, smoking, center, and average accelerometer wear time

*p<0.05; **p<0.01; ***p<0.001

Abbreviations: BMI, body mass index; cpm, counts per minute; LPA, light-intensity physical activity; MCS, mental component score; MVPA, moderate-to-vigorous intensity physical activity; PCS, physical component score; pts, points; ref., reference category; SB, stationary behavior.

**Supplemental Table 3. Association (std. β) of Baseline and Changes in MCS and PCS on Mean Total Activity, SB, LPA, and MVPA, Race/Sex Stratified Results**

|  |  | **Mean Total Activity (cpm)** | **SB (min/day)** | **LPA (min/day)** | **MVPA (min/day)** |
| --- | --- | --- | --- | --- | --- |
| **MCS** |  | *std. β* | *std. β* | *std. β* | *std. β* |
| *White Men (n=225)* | **Baseline**  **10-yr change** | **26.2***  **39.0***** | -10.0  **-16.7*** | 5.9  11.0 | **4.4***  **5.7**** |
| *Black Men (n=101)* | **Baseline**  **10-yr change** | 30.9  0.1 | **-28.0***  4.4 | **21.4***  -3.4 | 1.8  0.2 |
| *White Women (n=327)* | **Baseline**  **10-yr change** | 2.9  -2.4 | -4.1  -0.7 | 3.7  2.3 | -0.0  -0.8 |
| *Black Women (n=241)* | **Baseline**  **10-yr change** | 2.7  -7.5 | -0.3  3.0 | -2.0  -6.2 | 2.0  0.5 |
| **PCS** |  | std. β | std. β | std. β | std. β |
| *White Men (n=225)* | **Baseline**  **10-yr change** | **28.1****  16.0 | **-13.5***  -2.4 | 9.1  -1.2 | **5.2****  **3.3*** |
| *Black Men (n=101)* | **Baseline**  **10-yr change** | **38.3***  **40.6*** | **-27.8***  **-28.4*** | 20.2  **22.6*** | 5.3  **7.2*** |
| *White Women (n=327)* | **Baseline**  **10-yr change** | -0.3  8.3 | 1.5  0.6 | -2.9  -3.1 | 1.2  2.3 |
| *Black Women (n=241)* | **Baseline**  **10-yr change** | **30.8*****  **30.5***** | **-17.9****  **-17.1**** | **15.1***  10.3 | **3.5****  **4.0**** |

**Statistically significant results are bolded.**

Adjusted for baseline value of MCS or PCS, follow-up time, average accelerometer wear time, age, education, smoking, and center.

*p<0.05; **p<0.01; ***p<0.001

Abbreviations: BMI, body mass index; cpm, counts per minute; LPA, light-intensity physical activity; MCS, mental component score; MVPA, moderate-to-vigorous intensity physical activity; PCS, physical component score; pts, points; SB, stationary behavior.
