# Supplementary material for: Bidirectional associations of accelerometer-derived physical activity and stationary behavior with self-reported mental and physical health during midlife
Source: Int J Behav Nutr Phys Act. 2021 Jun 6;18:74. doi: 10.1186/s12966-021-01145-4 (PMC8180096; doi:10.1186/s12966-021-01145-4)
Supplement: Supplementary file 2 — Additional file 2: Supplemental Figure 1. Baseline and 10-year Follow-up Self-rated Health in CARDIA. [file 12966_2021_1145_MOESM2_ESM.docx]

**Supplemental Figure 1. Baseline and 10-year Follow-up Self-rated Health in CARDIA**

| 1. MCS   Baseline: 51.1 (8.8) pts  Follow-up: 52.2 (9.0) pts  Difference: +1.05 (9.07) pts; (p<0.001) | 1. PCS   Baseline: 52.2 (7.0) pts  Follow-up: 50.7 (8.1) pts  Difference: - 1.54 pts (7.30) (p<0.001) |
| --- | --- |

Data presented as mean (SD)
